# Supplementary material for: Tuberculosis-Related Hospitalizations in a Low-Incidence Country: A Retrospective Analysis in Two Italian Infectious Diseases Wards
Source: Int J Environ Res Public Health. 2019 Dec 23;17(1):124. doi: 10.3390/ijerph17010124 (PMC6981912; doi:10.3390/ijerph17010124)
Supplement: Supplementary file 1 [file ijerph-17-00124-s001.pdf]

Supplementary materials

Table S1. Descriptive analysis of the overall population.

**Variables**		***N* = 166**
Year of diagnosis, *n* (%)	2013	17 (10.2)
	2014	20 (12.1)
	2015	34 (20.5)
	2016	58 (34.9)
	2017	37 (22.3)
Median (IQR) age, years		37 (26–55)
Age >65 years		31 (18.7)
Age group, *n* (%)	0–24	31 (18.7)
	25–44	74 (44.6)
	45–64	30 (18.1)
	65–79	17 (10.2)
	≥80	14 (8.4)
Males, n (%)		118 (71.1)
Geographical area of origin, *n* (%)	Italy	52 (31.3)
	Africa	50 (30.1)
	Europe	42 (25.3)
	Other countries *	22 (13.3)
Immunodepression, n (%)		45 (27.1)
Causes of immunodepression, *n* (%)	HIV-positivity	14 (31.1)
	Haematological diseases	6 (13.3)
	Alcohol	4 (8.9)
	Diabetes mellitus	4 (8.9)
	Solid tumour	4 (8.9)
	Malnutrition	4 (8.9)
	Autoimmune disease	3 (6.7)
	Chronic renal failure	2 (4.4)
	Other diseases	4 (8.9)
Comorbidity, *n* (%)		39 (23.5)
TB form, *n* (%)	PTB **	95 (57.2)
	EPTB ***	61 (36.8)
	PTB and EPTB	10 (6.0)
**Clinical findings**		
Fever, *n* (%)		103 (62.4)
Cough, *n* (%)		89 (53.9)
Weight loss, *n* (%)		86 (52.1)
Haemoptysis, *n* (%)		21 (12.7)
Sweating, *n* (%)		47 (28.5)
Asthenia, *n* (%)		94 (57.0)
Dyspnoea, *n* (%)		40 (24.2)
Chest pain, *n* (%)		54 (32.7)
Median (IQR) duration of symptoms, weeks		8 (3–17)
**Diagnosis**		
Mantoux positivity, *n* (%)		89 (90.8)
IGRAs **** positivity, *n* (%)		132 (89.2)
Sputum smear positivity, *n* (%)		55 (61.8)
Sputum PCR positivity, *n* (%)		57 (65.5)

Sputum culture positivity, *n* (%)	37 (75.5)
Bronchoalveolar lavage smear microscopy, *n* (%)	22 (29.3)
Bronchoalveolar lavage PCR positivity, *n* (%)	48 (63.2)
Bronchoalveolar lavage culture positivity, *n* (%)	24 (61.5)
Other specimens, *n* (%)	22 (81.5)
Other specimens-smear microscopy positivity, *n* (%)	9 (33.3)
Other specimens-PCR positivity, *n* (%)	23 (85.2)
Other specimens-culture positivity, *n* (%)	4 (16.7)
Median (IQR) sputum conversion, weeks	3 (2–5)
**Radiological findings** *****	
Tree in bud, *n* (%)	31 (19.3)
Lung consolidation, *n* (%)	91 (56.5)
Cavities, *n* (%)	58 (36.0)
Nodular pattern, *n* (%)	67 (41.6)
Mediastinal lesions, *n* (%)	76 (47.2)
**TB drug resistance, *n*.16 (susceptibility test on 126 pts)**	
Phenotypic drug susceptibility (n. resistant 12/98)	
Resistance to pyrazinamide, *n* (%)	7 (58.3)
Resistance to isoniazid, *n* (%)	5 (41.6)
Resistance to rifampicin, *n* (%)	2 (1.6)
Resistance to streptomycin, *n* (%)	2 (1.6)
Resistance to ethambutol, *n* (%)	1 (0.8)
Xpert or Anyplex drug susceptibility (n. resistant 4/31)	
Resistance to isoniazid, *n* (%)	2 (50)
Resistance to rifampicin, *n* (%)	2 (50)
Resistance to aminoglycosides, *n* (%)	1 (25)
More than one resistance, n (%)	4 (25)
**TB therapy (data for 161 patients)**	
First-line therapy, *n* (%)	145 (90.1)
Initial second-line therapy, *n* (%) °	16 (9.9)
Drug modification, *n* (%)	24 (14.9)

* Asian and South American countries; ** PTB = pulmonary tuberculosis; *** EPTB = extrapulmonary tuberculosis; **** IGRAs = interferon gamma release assays; ***** data for 161 /166 patients; ° second-line therapy refers to the treatment of patients who started with one or more second-line drugs (other than rifampicin, isoniazid, pyrazinamide, ethambutol) in the composition of their TB therapy; IQR: interquartile range.
